# Methanol poisoning as a new world challenge: A review

**DOI:** 10.1016/j.amsu.2021.102445

**Published:** 2021-06-02

**Authors:** Zahra Nekoukar, Zakaria Zakariaei, Fatemeh Taghizadeh, Fatemeh Musavi, Elham Sadat Banimostafavi, Ali Sharifpour, Nasrin Ebrahim Ghuchi, Mahdi Fakhar, Rabeeh Tabaripour, Sepideh Safanavaei

**Affiliations:** a Department of Clinical Pharmacy, Faculty of Pharmacy, Mazandaran University of Medical Sciences, Sari, Iran; b Toxoplasmosis Research Center, Communicable Diseases Institute, Iranian National Registry Center for Lophomoniasis and Toxoplasmosis, Mazandaran University of Medical Sciences, Sari, Iran; c Toxicology and Forensic Medicine Division, Orthopedic Research Center, Imam Khomeini Hospital, Mazandaran University of Medical Sciences, Sari, Iran; d Psychiatry and Behavioral Sciences Center, Addiction Institute, Mazandaran University of Medical Sciences, Sari, Iran; e Pulmonary and Critical Care Division, Imam Khomeini Hospital, Iranian National Registry Center for Lophomoniasis (INRCL), Mazandaran University of Medical Sciences, Sari, Iran; f Knowledge and Information Science, Imam Khomeini Hospital, Mazandaran University of Medical Sciences, Sari, Iran

**Keywords:** Methanol, Metabolic acidosis, Blindness, Hemodialysis

## Abstract

**Background:**

Methanol poisoning (MP) occurs often via ingestion, inhalation, or dermal exposure to formulations containing methanol in base. Clinical manifestations of MP include gastrointestinal symptoms, central nervous system (CNS) suppression, and decompensated metabolic acidosis occurred with blurred vision and early or late blindness.

**Objective:**

This study reviewed the clinical manifestations, laboratory and radiology findings, and treatment approaches in MP.

**Discussion:**

Methanol is usually rapidly absorbed after ingestion and metabolized by alcohol dehydrogenase (ADH), then distributed to the body water to reach a volume distribution approximately equal to 0.77 L/kg. It is also eliminated from the body as unchanged parent compounds. Clinical manifestations of MP alone initiate within 0.5–4 h after ingestion and include gastrointestinal symptoms and CNS suppression. After a latent period of 6–24 h, depending on the absorbed dose, decompensated metabolic acidosis occurs with blurred vision and early or late blindness. Blurred vision with normal consciousness is a strong suspicious sign of an MP. The mortality and severity of intoxication are well associated with the severity of CNS depression, hyperglycemia, and metabolic acidosis, but not with serum methanol concentration. After initial resuscitation, the most important therapeutic action for patients with known or suspected MP is correction of acidosis, inhibition of ADH, and hemodialysis.

**Conclusion:**

Since MP is associated with high morbidity and mortality, it should be considered seriously and instantly managed. Delay in treatment may cause complications, permanent damage, and even death.

## Highlights

• We attempt to review clinical manifestations, laboratory and radiology findings, and treatment approaches in Methanol poisoning (MP).
• The most important therapeutic action for patients with known MP is correction of acidosis, inhibition of alcohol dehydrogenase, and hemodialysis.
• The review emphasizes that MP has a high morbidity and mortality rate and it must be taken seriously and treated immediatly.

## 1 Introduction

Methanol (CH3OH), which has long been used in mummification in ancient Egypt, was obtained from the distillation of wood, which in Greek roots was called methylene or wood wine [1]. It is a toxic alcohol used as a solvent or in denatured industrial alcohol. Methanol production reached industrial scale in 1923 and has found wide applications in various consumer industries, such as model cars, airplane fuel, perfumery, copy machine fluid, gas line antifreeze (“dry gas”), etc. [2].

Some factors contribute to the delay in receiving suitable care. First, in areas where alcohol consumption is unsocially or religiously acceptable, presentations may be delayed due to fear of punishment [3,4]. Second, the manifestations of early methanol toxicity are nonspecific, which leads to delay in diagnosis [5]. Clinical toxicologists encounter some limitations in diagnosing and treating methanol poisoning (MP) in Iran. Several teaching and referral hospitals have almost no laboratory amenities to evaluate blood levels of toxic alcohol and their metabolites, including methanol, formate, and formic acid concentrations, neither by gas chromatography with or without mass spectrometry confirmation (gold standard method) nor enzymatic assays. Furthermore, there are insufficient supplies and equipment to measure serum osmol and chloride for calculating anion gap and serum lactate level; even an arterial blood gases (ABG) test is performed on occasion. Although some of these problems may be due to the US sanctions against Iran, one should not ignore officials’ inattention to poisoned patients and maladministration [6]. Most of the MP cases reported in the United States involve ingestions of such products as windshield washer fluid, though most inhalational exposures involve carburetor cleaner.

The MP occurred via ingestion, inhalation, or dermal exposure with formulations containing methanol in base can also cause toxicity [7]. Reports show that most methanol toxicities are related to the ingestion of cologne and perfumes in Tunisia, Turkey, and India [8,9]. Herbal water possibly contain some level of alcohol impurity. Important factors influencing the amount of methanol and ethanol production include the duration of maceration and starting the distillation process, wood content of the plant, temperature, unopened bottles of distillates, soaking time, types of plants, the collection or storage of aromatic substances, and distillates pasteurization. Note that passing time will change the level of alcohol in distillates [[10], [11], [12], [13]].

A study in Hamadan, Iran showed that more than 50% of herbal drink samples were contaminated with methanol [14]. High concentrations of methanol and ethanol may cause toxicity in people taking herbal distillate products for a long time [15]. Following the coronavirus disease 2019 (COVID-19) pandemic in Iran (February 19, 2020 to April 27, 2020), there has been a significant increase in methanol-induced morbidity and mortality. This was the greatest prevalence of methanol mass poisoning in the country in recent periods. Because methanol is less expensive and more readily available than ethanol, some fraudsters in Iran use it instead of ethanol in home-made alcohol. Therefore, it is important to increase public knowledge about the deadly consequences of consuming fake alcohol sold on the black market [16,17]. Thus, the current study reviewed the clinical manifestations, laboratory and radiology findings, and treatment approaches in MP.

## 2 Discussion

### 2.1 TOXICOKINETICS/TOXICODYNAMICS

**Fig. 1:**
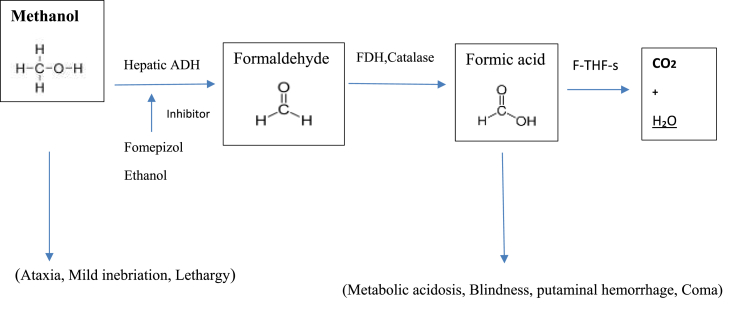
Methanol biotransformation.

The estimated least lethal dose of methanol for adults is almost 10 mL, though there are reports of consuming more than 400 mL without consequences [18,19]. Methanol is usually absorbed rapidly after ingestion, and undergoes first-pass hepatic effects and is metabolized by gastric alcohol dehydrogenase (ADH) [20] (Fig. 1). As mentioned before, there are three main ways to MP, and inhalation is not as common as others [21]. In chronic inhalation situations, the administration of folate and inhibiting ADH in the methanol metabolism pathway is preferred, but the need for hemodialysis varies from one case to the other [22,23]. Methanol penetrates throughout the skin; this route of intoxication is predominantly reported in infants even with fatal metabolic acidosis. Its toxic effects are associated with the duration of contact and some individual characteristics (large body surface-volume ratio) [[24], [25], [26]]. Methanol is distributed to the body water right after absorption to reach a volume equal to 0.77 L/kg. The methanol distribution half-life is about 8 min, which is longer than the absorption half-life; thus, the peak serum concentrations are achieved relatively fast after ingestion, and then fall [27]. Methanol can be eliminated from the body as unchanged parent compounds, and it has an insignificant renal excretion [28]. ADH and aldehyde dehydrogenase (ALDH) are the two key enzymes responsible for the oxidation of methanol by converting NAD + to NADPH to produce formic acid. Formate metabolites are bound by tetrahydrofolate and convert to water and CO2 by 10-formyltetrahydrofolate dehydrogenase [29].

### 2.2 Pathophysiology and clinical effects

**Fig. 2:**
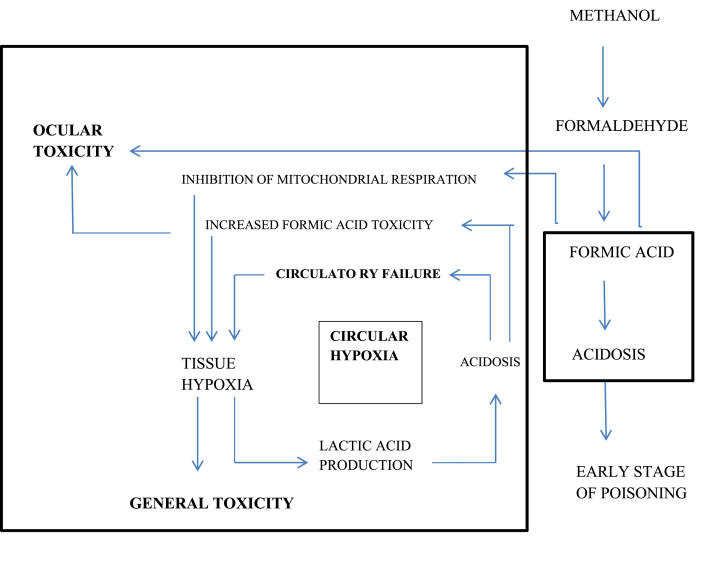
Mechanism of action in methanol poisoning.

Clinical manifestations of pure methanol toxicity initiate within 0.5–4 h after ingestion and include gastrointestinal disorders (nausea, vomiting, and abdominal pain) and central nervous system (CNS) suppression (confusion and drowsiness) (Fig. 2). Depending on the absorbed dose, after a latent period of 6–24 h, decompensated metabolic acidosis occurs with blurred vision, photophobia, diplopia, early or late blindness, and less commonly, nystagmus. Blurred vision with normal consciousness is a strong suspicious sign of an MP. Mortality and severity of clinical effects are well associated with the severity of CNS depression, hyperglycemia, and metabolic acidosis, but not with serum methanol concentration [18,[30], [31], [32]]. The pupils of MP patients are mydriatic, with a delayed or non-response to light.A high anion gap metabolic acidosis may be exposed at later phases of methanol. Hyperglycemia and hyperkalemia due to acidosis may also occur in MP [33]. Leukotriene (LT)-mediated neuro inflammation may show a significant role in the mechanisms of toxic brain injury in acute MP in patients. An important association between acute serum LT concentration and the result of poisoning may indicate the neuroprotective effect of a moderate increase in LT concentration observed in patients with MP [34].

#### 2.2.1 Neurologic manifestation

Depending on the dose, whole alcohol can cause inebriation and drunkenness.On a molar basis, it appears that alcohols with a higher molecular weight (isopropanol) are more poisonous than alcohols with a lower molecular weight (methanol) [35]. However, the absence of clear inebriation does not exclude ingestion, exclusively if the patient chronically drinks alcohol and is thereby tolerant to its CNS manifestation [36]. Additionally, serum methanol concentrations of 25–50 mg/dL may be accompanying toxicity, whereas in most conditions, one may legally drive an automobile with a blood alcohol concentration of up to 80 mg/dL. The CNS effects of MP are mediated through increased gamma-aminobutyric acid (GABA) –eric tone directly and through inhibition of presynaptic GABA (GABA-A receptors) and N-Methyl-d-aspartic acid (NMDA) glutamate receptors [[37], [38], [39], [40], [41]].

#### 2.2.2 Metabolic acidosis

One of the signs and symptoms of MP is metabolic acidosis with an elevated anion gap and osmol gap. This is a consequence of the breakdown of methanol to formate and formic acid. Since formic acid has no rapid natural metabolic pathway of elimination, it is accumulated.

#### 2.2.3 Visual impairment

Untreated methanol overdose causes specific ocular toxicity determined by destruction of optic nerve and pigmented retinal epithelial cells, resulting in visual defects, ranging from blurred vision to “snowfield vision” or total blindness in severe poisoning. Visual disorders are caused by formate metabolites and may occur up to 72 h after ingestion [42]. Vision loss may not be symmetric [43,44]. On visual field testing, central scotoma, hyperemia, pallor of the optic disc, papilledema, and an afferent papillary defect may be present, which are described as characteristic findings. Electroretinography may demonstrate a diminished b-wave [[45], [46], [47], [48]], a marker of bipolar cell dysfunction. Additionally, optical coherence tomography, which is similar to ultrasound but uses reflected light waves to image translucent tissues, may demonstrate peripapillary nerve fiber swelling and intraretinal fluid accumulation [49]. Formate is a mitochondrial toxin, inhibiting cytochrome oxidase and it thereby interferes with oxidative phosphorylation [[50], [51], [52]].

Although it is unclear why this results in ocular toxicity while other tissues are comparatively saved, retinal pigmented epithelial and optic nerve cells appear to be uniquely susceptible [48,53,54]. After several years of exposure, optic nerve atrophy, disc pallor, and severe cupping may be present, even with normal intraocular pressure [55].

#### 2.2.4 Brain impairment

**Fig. 3:**
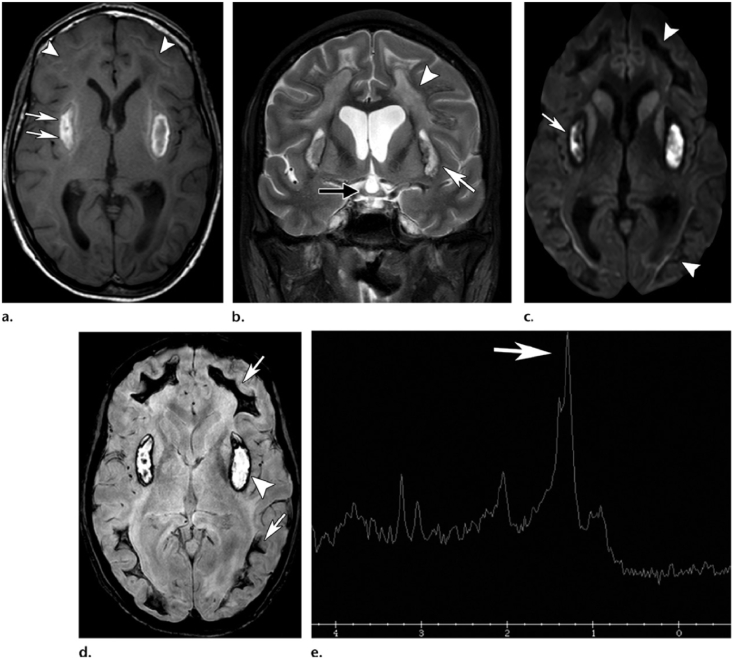
Basal ganglia and putamen involvement in methanol toxicity in MRI or CT scan view.

**Fig. 4:**
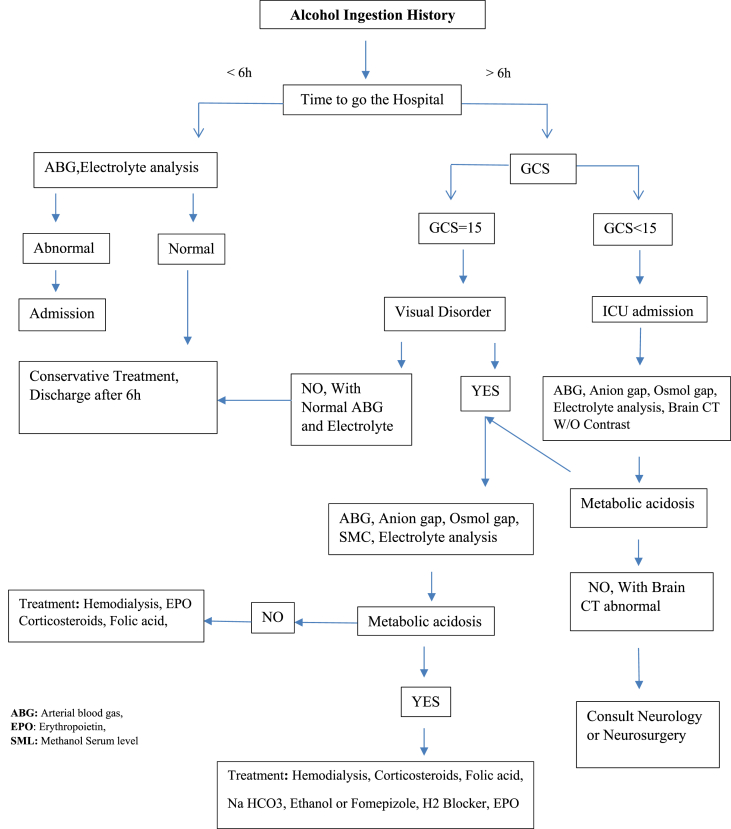
Algorithm of diagnosis and treatment methanol poisoning.

Bilateral basal ganglia lesions, bilateral necrosis of the putamen (with or without hemorrhage), and less commonly, the caudate nucleus is characteristically abnormal visualized on computed tomography (CT) scan or magnetic resonance imaging (MRI) after MP [[56], [57], [58], [59], [60], [61]] (Fig. 3). While these injuries are nonspecific and may occur in hypoxia due to other types of poisoning, in MP cases, they occur without hypoxia and hypotension due to direct toxic mechanism. Many patients develop Parkinsonism after poisoning by methanol, a finding that is consistent with the lesions in the basal ganglia lesions. In addition to clinical and laboratory findings, the existence of the putamen hemorrhage and insular sub cortex white matter necrosis is associated with a poor clinical outcome in patients with methanol toxicity [[62], [63], [64], [65], [66], [67], [68], [69], [70]] (Table 1). Both the retinal and neurological effects of MP may be permanent (see Fig. 4).

**Table 1:** Comparison of brain CT signs among survived and died patients with methanol poisoning.

CT findings	Survivors (n = 36)	Died (n = 6)	Significance
Putaminal necrosis	23 (63.9%)	4 (66.7%)	NS^a^
Putaminal hemorrhage	4 (11.1%)	3 (50%)	0.018^b^
Insular subcortical necrosis	3 (8.3%)	3 (50%)	0.007^b^
Insular subcortical hemorrhage	1 (2.8%)	0	NS^a^
Frontal white matter necrosis	5 (13.9%)	2 (33.3%)	NS^b^
Occipital white matter necrosis	3 (8.3%)	2 (33.3%)	NS^a^
Forth ventricle hemorrhage	0	1 (16.7%)	NS^a^
Generalized supratentorial necrosis	0	1 (16.7%)	NS^a^

NS = not significant.

a The Fisher's exact test.

b The Chi-square test.

According to the results of two studies on MP outbreaks in Estonia and Iran, predicting the long-term outcomes of retinal and neurologic sequelae of methanol toxicity is difficult [31,[71], [72], [73], [74], [75], [76]].

#### 2.2.5 Kidney impairment

Rarely, injury to other tissues may also occur. Both acute kidney injury (AKI) and pancreatitis are reported after MP [71,[75], [76], [77], [78], [79]]. Some of the AKI cases resulting from MP may be due to myoglobinuria [80]. AKI is associated with more severe poisoning, as manifested by low initial serum pH, high initial osmolality, and high peak formate concentration [81].

#### 2.2.6 Liver impairment

Pathologic abnormalities of the liver, esophagus, and gastric mucosa are also found in some fatal MP cases. Histological changes in liver tissue include microand macrovesicular steatosis, central hepatocyte necrosis, mild intrahepatic bile stasis, and fluffy and hydropic degeneration. Liver impairment occurred in males 6.3 times more than females, and most of the victims were middle-aged people [82,83].

## 3 Diagnostic testing

### 3.1 Methanol and metabolite concentrations

Serum methanol and formate concentrations are ideal tests when toxic alcohol poisoning is suspected a few hours after exposure. However, these concentrations are most commonly measured by gas chromatography or enzymatic assays for methanol and formate, and may need more readily available clinical tests [[84], [85], [86]]. A group in Finland defined a respiratory test for methanol, using a portable Fourier transform infrared (FT-IR) analyzer similar to the “breathalyzer” used by legal agents [87].

A formate concentration may be important in the case of late presentation after methanol ingestion. In one study, formate was detected in blood samples from 97% of patients who died of MP; all of these patients had detectable blood or vitreous methanol concentrations [[88], [89], [90]]. Traditionally, a methanol concentration greater than 25 mg/dl has been considered toxic, but the evidence supporting this as a threshold is often questioned. Almost all reported cases of MP involve patients with delayed presentations who already have metabolic acidosis. The information currently available is insufficient to apply a 25 mg/dl management threshold in a patient presenting early after ingestion without acidosis [91].The primary laboratory tests should include serum electrolytes (Na, K, BS, BUN, Cr, U/A, and plasma osmolality) and serum ethanol and methanol concentrations. Blood gas analysis with a lactate concentration is also useful in the early assessment of seemingly ill patients.

### 3.2 Osmol gap & anion gap

When patients are presented with an anion gap acidosis of unknown causation and mostly with no history of ingestion, the first suspicion is often the possibility of methanol toxicity. Unless clinical evidence suggests otherwise, it is significant to exclude metabolic acidosis with ketoacidosis and elevated lactate concentration, which are the most common causes of anion gap acidosis, before pursuing methanol in these patients [92]. {AG: Na – (cl + Hco3 (= 12–14, OG: OC = 2Na + Glu/18 +Bun/2.8}

The acidosis takes time to develop, sometimes up to 16–24 h for methanol. Thus, the absence of an initial anion gap raised after described methanol consumption does not exclude the diagnosis. A potential early replacement sign of an MP is an elevated osmol gap. However, a raised osmol gap is neither sensitive nor specific for MP [[93], [94], [95], [96], [97]].

As methanol is metabolized to organic acid anions, the anion gap is elevated whereas the osmol gap is decreased; thus, patients who present to the emergency room primarily after consumption may have a high osmol gap and normal anion gap, while those who come later may have the reverse [98,99].

One retrospective and one prospective study attempted to evaluate the performance of the osmol gap as a diagnostic test; in both cases, the osmol gap performed fairly well. However, the sample size of both studies was small (20 patients with toxic alcohol poisoning in the retrospective study and 28 patients with MP in the prospective study). The prospective study identified three patients with significant poisoning and acidosis but “normal” osmol gaps, which was defined as less than 25 in the study [98,100].

### 3.3 Lactate concentration

MP by producing formate, as an inhibitor of oxidative phosphorylation and induction of anaerobic metabolism, can cause raised lactate concentrations. Additionally, in severely intoxicated patients, hypotension and organ failure can also produce elevated lactate concentration. However, lactate production by these mechanisms results in serum concentrations not greater than 5 mmol/L.

### 3.4 Other diagnostics

Blood sugar concentration is obtained as part of routine laboratory analysis. Hyperglycemia was associated with a higher risk of death after MP in one retrospective study, with an odds ratio of 6.5.However, this has not been prospectively validated yet [32]. Patients with abdominal pain should also be tested for liver function and serum lipase because of the possibility of associated hepatitis and pancreatitis. Although brain CT and MRI disorders are frequently reported in MP, in the absence of neurological disturbances on physical examination, routine CT scans and MRIs are not indicated.

### 3.5 Prognostic and diagnostic testing

Increases in both anion gap and osmol gap and evaluation of blood gas may be useful for risk stratification in MP. A review of reported toxic alcohol cases identified risk factors for morbidity and mortality in patients with MP. According to the results, no patient with a pH greater than 7.22 and anion gap less than 30 meq/l or osmolar gap less than 49 died. A pH less than 7.22 was an even better predictor of mortality [101]. Another retrospective study of risk factors for poor outcomes in MP found that only pH less than 7.00 (also coma or a >24-h delay to presentation) was associated with death [102].

Similarly, another study examined the markers for poor visual outcome after MP and found that pH was the best predictor, with the amount greater than 7.20 associated with a high possibility of only transient visual sequelae [103].

The factors suggesting a poor prognosis for MP at the admission time include grade of metabolic acidosis, high serum methanol levels or the long time passed from ingesting, loss of consciousness, seizures, respiratory arrest, and increased PCO2 in the severely acidotic patients [[104], [105], [106], [107], [108], [109], [110], [111], [112]]. In critically ill patients due to methanol toxicity, the mechanisms of hyperglycemia are acute pancreatitis and stress-induced hyperglycemia [[113], [114], [115], [116], [117]].

There was no relationship between electrocardiogram (ECG) variations, time between consumption, and treatment in one retrospective observational case series. Although cardiotoxicity was high in MP, no ECG changes could predict mortality. These findings do not rule out the need to routinely run ECG for cardiotoxicity in every single patient intoxicated with methanol [118].

## 4 Treatment

Methanol may cause coma and respiratory arrest; thus, intubation and mechanical ventilation are required for patients with severe poisoning. Methanol frequently causes hypotension by vasodilation and vomiting, and many patients require hydration with intravenous crystalloid. Since methanol has rapid absorption and limited binding to activated charcoal, gastrointestinal decontamination is rarely indicated [20].

The most significant part of beginning treatment in patients with known or suspected MP is the blockade of ADH, which prevents the formation of toxic metabolites [119,120].

### 4.1 Ethanol

Ethanol (1 ml/kg of 5-fold diluted alcohol 96 as the loading dose, 0.16 ml/kg/h as the maintenance dose orally or by NGT, or ethanol 10%: 10 ml/kg IV as the loading dose, then 1 ml/kg/h up to ethanol serum level 150 mg/dl) is the common method of ADH inhibition and may still be the only choice in some institutions. The side effects of infusion include hypotension, respiratory depression, flushing, hypoglycemia, pancreatitis, gastritis, and inebriation; hence, patients receiving intravenous (IV) ethanol require admission to an intensive care unit (ICU). The true incidence of these complications is unclear. In one study, it was shown that complications of ethanol infusion in children were uncommon [121]. However, in another review of 49 adults treated with ethanol infusions, 92% of patients had at least one adverse event [122]. When intensive monitoring is unavailable, prescribing ethanol orally is also effective, especially when the patient is taken to hospital late.

### 4.2 Fomepizole

Fomepizole (4-methylpyrazole 15 mg/kg as the loading dose, 10 mg/kg as the maintenance every 12 h up to 4 doses, and then 15 mg/kg every 12 h up to the serum methanol concentration below 25 mg/dl) is a competitive antagonist of ADH that has many advantages over ethanol. When fomepizole is administered as an IV bolus every 12 h, monitoring is not needed as with an ethanol infusion. Since it does not cause inebriation and is associated with fewer side effects, it does not need ICU monitoring. Hence, despite being extremely expensive, it is preferred to ethanol [104,[123], [124], [125], [126], [127]]. Additionally, the cost difference will vary depending on the setting of poisoning and the healthcare delivery system of the country. A study in Belgium discovered that treating with ethanol and dialysis was significantly less expensive than fomepizole alone [128]. Bradycardia and hypotension may occur after fomepizole infusion; thus, vital signs should be monitored carefully during and after each dose [129,130]. A review of reported cases in which fomepizole was used in children suggested that it is safe and effective with weight-based dosing as in adults [131]. Pharmacokinetic data show that there is no significant difference in serum concentrations between oral and IV fomepizole [132]. Indications for fomepizole or ethanol therapy could be based on clinical and laboratory findings. Every patient with a credible history of methanol consumption and a high anion gap acidosis without another description or an obviously elevated osmol gap should be treated.

### 4.3 Abacavir

Abacavir (antiretroviral drug) is a substrate for ADH, which delays the metabolism of methanol. It has been suggested that abacavir could have potential efficacy as an alternative to fomepizole in places where fomepizole is unavailable.

### 4.4 H2 blockers

H2 blockers are inhibitors of gastric and hepatic ADH and could cause improved pH, formate concentrations, and retinal histopathology [[133], [134], [135]].

### 4.5 Hemodialysis

Hemodialysis removes all parent toxic alcohols and their metabolites during the first few hours after dialysis; redistribution of methanol could elevate methanol concentrations [136]. Hemodialysis clears both methanol and its toxic metabolites from the blood and corrects the acid–base disturbance. The indications for hemodialysis have become more restricted by the onset of fomepizole because of its effectiveness combined with its low incidence of adverse effects.

Even a patient with a moderately elevated serum methanol concentration (80 mg/dL or 2.5 mmol/L) was successfully treated with fomepizole alone [137]. Based on toxicokinetic data, some patients might be treated with or without delayed dialysis, particularly in epidemic scenarios where the need for hemodialysis may exceed the availability [138]. Although formate is normally cleared rapidly once ADH is blocked, the half-life increases with higher serum methanol concentrations and varies from 2.5 to 12.5 h. In severe poisoning cases, formate was eliminated at a slow rate with a half-life of 77 h until hemodialysis was initiated, underscoring the importance of hemodialysis in patients with significant metabolic acidosis [[139], [140], [141]].

Thus, indications for hemodialysis include metabolic acidosis and signs of end-organ damage (Table 2).

**Table 2:** Indications Hemodialysis in Methanol poisoning.

1	Persistence metabolic acidosis: PH < 7.25, anion gap >30mEq/l
2	Signs of end-organ damage (Visual, CNS and Renal abnormality)
3	Deteriorating vital signs despite conservative therapy
4	Electrolyte abnormality
5	Methanol serum level >50 mg/dl

According to the data extracted from one case series, an increase in formate concentration was a better predictor of clinically significant poisoning than methanol concentrations [142].

Although the elimination of formate by hemodialysis is considerable, the whole clearance did not seem to increase significantly above endogenous clearance in patients treated with folate and bicarbonate [[143], [144], [145]].

Many patients will need several cycles of hemodialysis to excrete methanol. Nephrologists determine the time required for dialysis [[146], [147], [148], [149], [150]].

Continuous renal replacement therapy (CRRT), such as continuous venovenous hemodiafiltration (CVVH), has sometimes been used in patients with MP. Hemodialysis is much more effective in removing drugs than CRRT, and it is approximately the preferred modality if available. However, if there is a contraindication to hemodialysis, such as hemodynamic instability or severe cerebral edema, or if hemodialysis is unavailable, CRRT may be considered an intervention that may offer some advantages over no extracorporeal removal at all. A pharmacokinetic model revealed that the addition of CRRT could decrease the duration of treatment by 40% [119,151].

### 4.6 Effects of extracorporeal treatment (ECTR) on clinical results

Despite the biases and limitations of the accessible clinical documents, the results of some reviews were considered possibly useful for guiding judgments about ECTR in MP. For example, they presented some evidence supporting the results of ECTR in improving acidosis and visual disorder. However, this study is not essentially autonomous of the effect of co-administered antidote treatment. Additionally, visual deficiency recovered in some patients who did not receive ECTR but were managed with fomepizole and ethanol [105,[152], [153], [154]]. Empirical studies in canines also showed that control animals developed neuronal symptoms that consequently improved without ECTR [155]. Since primary neuronal injury (including vision deficits) was not completely recorded in some cases, clinical benefits cannot be easily assessed, partly due to a change in the level of consciousness at the time of exposure. In the absence of severe toxicity by balancing, the decision to use ECTR is determined [152].

In situations to prevent the formation of formate, ethanol or fomepizole therapy has been quickly started, and if there are no acute clinical signs, ECTR does not need to be initiated immediately. Instead, this can be initiated at a later time until transfer to a center with ECTR services or sufficient time for ECTR staff to arrive [152].

### 4.7 Adjunctive treatment

There are many therapeutic adjuncts to ADH inhibitors with or without hemodialysis that should be considered for these patients.

#### 4.7.1 Folate and leucovorin

Folate and leucovorin (folic acid 1 mg/kg up to 50 mg every 4 h or folinic acid IV in D.W 5% in 30–60 min, then 10 mg daily up to 1 month) in animal models enhanced the clearance of formate and formic acid; however, there is only one human case report displays enhanced formate elimination with Folic Acid therapy [[156], [157], [158]]. Formate is less toxic than undissociated formic acid. Formic acid has a much higher affinity for cytochrome oxidase in the mitochondria, the terminal purpose site for toxicity. Formate can also diffuse into the target tissues [159]. It is also recommended to prescribe vitamin B12 100 mg and vitamin B6 100 mg daily for up to 1 month [160].

#### 4.7.2 Alkalinization

Alkalinization by NaHCO3 infusion shifts the balance toward the less toxic and dissociated form, in accordance with the Henderson–Hasselbalch equation. Data from uncontrolled case series demonstrate that patients treated with bicarbonate alone had better than expected results after severe methanol toxicity [111], but the outcomes are equivocal in patients also treated with ADH inhibitor and hemodialysis [106,161]. However, in the absence of contraindications to bicarbonate infusion, alkalinization should be used in patients with suspected MP and considerable acidosis, and pH more than 7.20 is a reasonable endpoint.

#### 4.7.3 Corticosteroids

Corticosteroids (methylprednisolone 500 mg q 12 h IV up to 5 days, then prednisolone 1 mg/kg up to 2 weeks) are useful for retinal injury following MP. In an uncontrolled case series, 13 of 15 patients showed improvement in their vision after treatment with 1-g methylprednisolone daily for 3 days, with one having worsening vision and one unchanged [162]. Another uncontrolled case series used a slightly different dosing regimen, with 250 mg of intravenous methylprednisolone administered every 6 h then prednisolone 1 mg/kg daily for 10 days. After treatment, visual acuity improved, but methanol concentrations were not reported; since exposure was not confirmed and the acuity of data was not reported for individual patients, it was unclear whether there was any worsened case [163,164]. Another series of four patients with mild MP were given the same treatment regimen, and the results showed some improvement in vision [165]. During the MP outbreak in India in 2009, all 63 male patients with evidence of optic neuritis (at least 60% of 46 survivors) were treated with retro-bulbar injections of triamcinolone, and 75% had some improvement [166]. However, these documents are inadequate to support the routine usage of corticosteroids in MP [167].

#### 4.7.4 Erythropoietin

In vitro and animal studies have shown the neuroprotective effect of erythropoietin **(**EPO **(**10.000 U amp Eprex q 12 h up to 3 days if Hb < 16, SBP<160 mmHg) against hypoxic damage [160,168,169]. The proposed mechanisms include direct neuroprotection, anti-apoptotic, anti-inflammatory, and anti-oxidant effects and improving blood flow to the injured tissue [[170], [171], [172], [173]]. Intravenous administration of EPO combined with IV methylprednisolone followed by oral prednisolone 1 mg/kg up to two weeks resulted in an effective treatment for methanol-induced toxic optic neuropathy [160].

Another study shows that intravenous EPO causes a relatively rapid increase in visual acuity when used within 3 weeks of methanol ingestion. EPO may be a promising modality of treatment for methanol-associated optic neuropathy [174].

## 5 Special populations

### 5.1 Pregnancy

There are few reported cases of pregnant women with toxic alcohol poisoning, but some conclusions can be drawn from the available data. Methanol readily crosses the placenta and perinatal maternal methanol ingestion has resulted in the death of a newborn [175].

### 5.2 Children

In younger children, a common clinical manifestation occurs when a child swallows one or two amounts of a concentrated methanol solution. These children should be transferred to the hospital [176].

Most cases of unintentional exposure evaluated shortly after consumption does not manifest significant acidosis or any evidence of organ damage. The ideal assessment is to estimate the serum alcohol concentration to absorbed dose and predict the clinical course. Unfortunately, such concentrations within a few hours of consumption are rarely accessible, creating a diagnostic and therapeutic problem. If the ingestion is accidental and of small volume, the product is exactly identified, the patient is without symptoms, the patient has a normal pH and anion gap, there is no co-ingestion or treatment with ethanol or fomepizole, the clinician can observe the patient and monitor blood gases and electrolytes every one to 2 h to exclude the development of a metabolic acidosis or an increasing anion gap. If acidosis does not develop within 8 h of the ingestion, the MP risk is excluded. ADH inhibition should not be initiated during this observation period as such treatment may prevent the development of acidosis even in patients with massive ingestions. In other words, the co-ingestion of ethanol or treatment with ADH inhibitor invalidates the above protocol. Parents should be counseled regarding safe storage of household products and pharmaceuticals before discharge. In symptomatic pediatrics, there is limited data supporting that fomepizole is safe and effective in children, and the dosing protocol is similar to that of adults [177]. Hemodialysis, hydration, and bicarbonate administration are rational approaches for managing methanol overdose in children [178].

## 6 Conclusion

Acute alcohol poisoning has high morbidity and mortality, and needs to be considered seriously and instantly managed. Delay in treatment may cause complications, permanent damage, or death. Since MP is associated with high morbidity and mortality, if there is a history of suspected alcohol ingestion or damage to vital organs, the patient should be hospitalized and appropriate diagnostic and therapeutic management should be performed. Moreover, if available, a clinical toxicologist should be consulted to prevent irreversible damage.

## Ethical approval

The study was approved by our local ethics committee.

## Sources of funding

This study was not funded.

## Author contribution

ZZ proposed the study, ZN wrote the first draft of this work. MF, FT, Ash and SS critically reviewed the manuscript. NEG, FM RT were involved in searching and data extraction. All authors read and approved the final version of the manuscript.

## Trial registry number

Our work is a review article.

## Guarantor

Zakaria Zakariaei.

## Consent

None.

## Article summary

1. Why is this topic important?

Due to the high incidence of methanol poisoning in Iran and many other countries around the world, this study was designed to review the basic knowledge and new findings of different aspects of methanol toxicity.

2. What does this review attempt to show?

This review attempts to determine the clinical manifestations, laboratory and imaging findings regarding methanol toxicity and effective treatment options.

3. What are the key findings?

The key findings include clinical manifestations (gastrointestinal symptoms, central nervous system suppression, and ocular toxicity determined by destruction of optic nerve and blindness), laboratory findings (metabolic acidosis with elevated anion gap and osmol gap), and radiology findings (bilateral basal ganglia lesions and bilateral necrosis of the putamen).

4. How is patient care impacted?

Using early and effective treatment strategies can prevent the acute and late complications of methanol poisoning**.**

## Declaration of competing interest

None.

## References

[1] Ott J., Gronemann V., Pontzen F. (2000 Jun 15). Methanol. Ullmann’s Encyclopedia of Industrial Chemistry.

[2] Katz K.D., Ruha A.M., Curry S.C. (2004 Nov 1). Aniline and methanol toxicity after shoe dye ingestion. J. Emerg. Med..

[3] Rostrup M., Edwards J.K., Abukalish M. (2016). The methanol poisoning outbreaks in Libya 2013 and Kenya 2014. PloS One.

[4] Hassanian-Moghaddam H., Nikfarjam A., Mirafzal A. (2015). Methanol mass poisoning in Iran: role of case finding in outbreak management. J. Public Health.

[5] Rostrup M., Edwards J.K., Abukalish M. (2016). The methanol poisoning outbreaks in Libya 2013 and Kenya 2014. PloS One.

[6] Banagozar Mohammadi A., Delirrad M. (2019 May 1). Problems with methanol poisoning outbreaks in Iran. Alcohol Alcohol.

[7] Hassanian-Moghaddam H., Nikfarjam A., Mirafzal A. (2015 Jun 1). Methanol mass poisoning in Iran: role of case finding in outbreak management. J. Publ. Health.

[8] Brahmi N., Blel Y., Abidi N. (2007 Jan 1). Methanol poisoning in Tunisia: report of 16 cases. Clin. Toxicol..

[9] Kalkan S., Cevik A.A., Cavdar C. (2003 Dec). Acute methanol poisonings reported to the drug and poison information center in izmir, Turkey. Vet. Hum. Toxicol..

[10] Laats M.M., Grosdenis F., Recourt K. (1997). Partial purification and characterization of pectin methylesterase from green beans (Phaseolus Vulgaris L.). J. Agric. Food Chem..

[11] Anthon G.E., Barrett D.M. (2006). Characterization of the temperature activation of pectin methylesterase in green beans and tomatoes. J. Agric. Food Chem..

[12] Bouchard M., Droz P.O., Carrier G. (2001). A bio-logically based dynamic model for predicting the disposition of methanol and its metabo-lites in animals and humans. Toxicol. Sci..

[13] Cabaroglu T. (2005). Methanol contents of Turk-ish varietal wines and effect of processing. Food Contr..

[14] Nili-Ahmadabadi A., Sedaghat M., Ranjbar A. (2016). Quantitative analysis and health risk assessment of methanol in medicinal herbal drinks marketed in Hamadan, Iran. J. Appl. Pharmaceut. Sci..

[15] Yousefi M., Afshari R., Sadeghi M., Salari R. (2018 Jun). Measurement of methanol and ethanol contents in most commonly used herbal distillates produced by three Famous Brands. Iran. J. Public Health.

[16] Soltaninejad K. (2020 Jul). Methanol mass poisoning outbreak, a consequence of COVID-19 pandemic and misleading messages on social media. Int. J. Occup. Environ. Med..

[17] Shokoohi M., Nasiri N., Sharifi H. (2020 Jun 4). A Syndemic of COVID-19 and Methanol Poisoning in Iran: Time for Iran to Consider Alcohol Use as a Public Health Challenge? Alcohol (Fayetteville, Ny).

[18] Paasma R., Hovda K.E., Hassanian-Moghaddan H. (2012). Risk factors related to poor outcome after methanol poisoning and the relation between outcome and antidotes – a multicenter study. Clin. Toxicol..

[19] Hassanian-Moghaddam H., Noroozi A., Balali-Mood M. (2009). Clinical Guideline for Treatment of Methanol Poisoning.

[20] Elwell R.J., Darouian P., Bailie G.R. (2004). Delayed absorption and postdialysis rebound in a case of acute methanol poisoning. AJEM (Am. J. Emerg. Med.).

[21] Bebarta V.S., Heard K., Dart R.C. (2006 Oct 1). Inhalational abuse of methanol products: elevated methanol and formate levels without vision loss. Am. J. Emerg. Med..

[22] LoVecchio F., Sawyers B., Tholel D. (2004 Oct). Outcomes following abuse of methanol containing carburetor cleaners. Hum. Exp. Toxicol..

[23] Givens M., Kalbfleisch K., Bryson S. (2008). Comparison of methanol exposure routes reported to Texas poison control centers. West. J. Emerg. Med..

[24] Oguz A.B., Gunalp M., Polat O. (2019 Nov 1). Transdermal methanol intoxication. Arch. Iran. Med..

[25] Darwish A., Roth C.E., Duclos P. (2002 Oct 4). Investigation into a cluster of infant deaths following immunization: evidence for methanol intoxication. Vaccine.

[26] Bal Z.S., Can F.K., Anil A.B. (2016 Aug 1). A rare cause of metabolic acidosis: fatal transdermal methanol intoxication in an infant. Pediatr. Emerg. Care.

[27] Graw M., Haffner H.T., Althaus L. (2000). Invasion and distribution of methanol. Arch. Toxicol..

[28] Kraut J.A., Jurtz I. (2008). Toxic alcohol ingestions: clinical features, diagnosis, and management. Clin. J. Am. Soc. Nephrol..

[29] Pietzke M., Meiser J., Vazquez A. (2020 Mar 1). Formate metabolism in health and disease. Molecular metabolism.

[30] Wiener S.W., Nelson L.S. (2011). Toxic alcohols. Goldfrank's Toxicologic Emergencies.

[31] Sanaei-Zadeh H., Zamani N., Shadnia S. (2011). Outcomes of visual disturbances after methanol poisoning. ClinToxicol (Phila).

[32] Sanaei-Zadeh H., Esfeh S.K., Zamani N. (2011). Hyperglycemia is a strong prognostic factor of lethality in methanol poisoning. J. Med. Toxicol..

[33] Wiener S.W., Nelson L.S. (2015). Toxic alcohols. Goldfrank's Toxicologic Emergencies.

[34] Zakharov S., Kotikova K., Nurieva O. (2017 Apr 21). Leukotriene-mediated neuroinflammation, toxic brain damage, and neurodegeneration in acute methanol poisoning. Clin. Toxicol..

[35] Wallgren H. (1960). Relative intoxicating effects on rats of ethyl, propyl and butyl alcohols. Acta Pharmacol Toxicol Toxicol.

[36] Symington L., Jackson L., Klaassen (2005). Toxic alcohol but not intoxicated—a case report. Scot. Med. J..

[37] Ariswodola O.J., Weiner J.L. (2004). Ethanol potentiation of GABAergic synaptic transmission may be self-limiting: role of presynaptic GABAB receptors. J. Neurosci..

[38] Carta M., Mameli M., Valenzuela C.F. (2004). Alcohol enhances GABAergic transmission to cerebellar granule cells via an increase in Golgi cell excitability. J. Neurosci..

[39] Grobin A.C., Matthews D.B., Devaud L.L., Morrow A.L. (1998). The role of GABAA receptors in the acute and chronic effects of ethanol. Psychopharmacologia.

[40] Hoffman P.L. (2003). NMDA receptors in alcoholism. Int. Rev. Neurobiol..

[41] Mihic S.J. (1999). Acute effects of ethanol on GABAA and glycine receptor function. Neurochem. Int..

[42] Newman N, Biousse V (2014). Diagnostic approach to vision loss. CONTINUUM: Lifelong Learning in Neurology.

[43] Chung T.N., Kim S.W., Park Y.S., Park I. (2010). Unilateral blindness with third cranial nerve palsy and abnormal enhancement of extraocular muscles on magnetic resonance imaging of orbit after the ingestion of methanol. Emerg. Med. J..

[44] Lu J.J., Kalimullah E.A., Bryant S.M. (2010). Unilateral blindness following acute methanol poisoning. J. Med. Toxicol..

[45] Bennett I.L., Cary F.H., Mitchell G.L., Cooper M.N. (1953). Acute methyl alcohol poisoning: a review based on experiences in an outbreak of 323 cases. Medicine.

[46] Onder F., Ilker S., Kansu T. (1999). Acute blindness and putaminal necrosis in methanol intoxication. Int. Ophthalmol..

[47] Ziegler S.L. (1921). The ocular menace of wood alcohol poisoning. J. Am. Med. Assoc..

[48] Treichel J.L., Murray T.G., Lewandowski M.F. (2004). Retinal toxicity in methanol poisoning. Retina.

[49] Fujihara M., Kikuchi M., Kurimoto Y. (2006). Methanol-induced retinal toxicity patient examined by optical coherence tomography. Jpn. J. Ophthalmol..

[50] Erecinska M., Wilson D.F. (1980). Inhibitors of cytochrome c oxidase. Pharmacol. Ther..

[51] Nicholls P. (1976). The effect of formate on cytochrome aa3, and on electron transport in the intact respiratory chain. Biochim. Biophys. Acta.

[52] Nicholls P. (1975). Formate as an inhibitor of cytochrome c oxidase. Biochem. Biophys. Res. Commun..

[53] Treichel J.L., Henry M.M., Skumatz C.M.B. (2003). Formate, the toxic metabolite of methanol, in cultured ocular cells. Neurotoxicology.

[54] Eells J.T., Henry M.M., Lewandowski M.F. (2000). Development and characterization of a rodent model of methanol-induced retinal and optic nerve toxicity. Neurotoxicology.

[55] Shin Y.W., Uhm K.B. (2011). A case of optic nerve atrophy with severe disc cupping after methanol poisoning. Kor. J. Ophthalmol..

[56] Ahsan H., Akbar M., Hameed A. (2009). Diffusion weighted image (DWI) findings in methanol intoxication. J. Pakistan Med. Assoc..

[57] Almansori M., Ahmed S.N. (2007). CT findings in methanol intoxication. CMAJ (Can. Med. Assoc. J.).

[58] Arora V., Nijjar I.B., Multani A.S. (2007). MRI findings in methanol intoxication: a report of two cases. Br. J. Radiol..

[59] Dujardin M., Peeters E., Ernst C., Stadnik T. (2006). Bilateral putaminal necrosis due to methanol abuse. JBR-BTR..

[60] Jarwani B.S., Motiani P., Divetia R., Thakkar G. (2012). Rare combination of bilateral putaminal necrosis, optic neuritis, and polyneuropathy in a case of acute methanol intoxication among patients met with hooch tragedy in Gujarat, India. J. Emergencies, Trauma, Shock.

[61] Sanaei-Zadeh H. (2012). Typical bilateral putaminal lesions of methanol intoxication. J. Emerg. Med..

[62] McLean D.R., Jacobs H., Mielke B.W. (1980). Methanol poisoning: a clinical and pathological study. Ann. Neurol..

[63] Guggenheim M.A., Couch J.R., Weinberg W. (1971). Motor dysfunction as permanent complication of methanol ingestion. Arch. Neurol..

[64] Massoumi G., Saberi K., Eizadi-Mood N. (2012). Methanol poisoning in Iran, from 2000 to 2009. Drug Chem. Toxicol..

[65] Reddy N.J., Lewis L.D., Gardner T.B. (2007). Two cases of rapid onset Parkinson's syndrome following toxic ingestion of ethylene glycol and methanol. Clin. Pharmacol. Ther..

[66] Keleş G.T., Örgüç S., Toprak B. (2007). Methanol poisoning with necrosis corpus callosum. Clin. Toxicol..

[67] Askar A., Al-Suwaida A. (2007). Methanol intoxication with brain hemorrhage: catastrophic outcome of late presentation. Saudi J Kidney Dis Transplant.

[68] Sebe A., Satar S., Uzun B. (2006 Dec 1). Intracranial hemorrhage associated with methanol intoxication. MSJM (Mt. Sinai J. Med.).

[69] Paliwal V.K., Uniyal R., Azim A. (2016 Sep). Haemorrhagic putaminal necrosis, optic atrophy and coma: a triad suggestive of methanol poisoning. Anaesth. Intensive Care.

[70] Taheri M.S., Moghaddam H.H., Moharamzad Y. (2010 Feb 1). The value of brain CT findings in acute methanol toxicity. Eur. J. Radiol..

[71] Hantson P., Mahieu P. (2000). Pancreatic injury following acute methanol poisoning. J. Toxicol. Clin. Toxicol..

[72] Blanco M., Casado R., Vazquez F., Pumar J.M. (2006). CT and MR imaging findings in methanol intoxication. Am. J. Neuroradiol..

[73] Hegde A.N., Mohan S., Lath N., Lim C.T. (2011). Differential diagnosis for bilateral abnormalities of the basal ganglia and thalamus. Radiographics.

[74] Sefidbakht S., Rasekhi A.R., Kamali K. (2007). Methanol poisoning: acute MR and CT findings in nine patients. Neuroradiology.

[75] Peters A.S., Schwarze B., Tomandl B. (2007). Bilateral striatal hyperintensities on diffusion weighted MRI in acute methanol poisoning. Eur. J. Neurol..

[76] Hlusicka J., Mana J., Vaneckova M. (2020). MRI-based brain volumetry and retinal optical coherence tomography as the biomarkers of outcome in acute methanol poisoning. Neurotoxicology.

[77] Korchanov L.S., Lebedev F.M., Lizanets M.N. (1970). Treatment of patients with acute kidney insufficiency caused by methyl alcohol poisoning. Urol. Nefrol..

[78] St, Wang Y.T., Hou Y.C. (2019 Dec 1). Acute kidney injury and the risk of mortality in patients with methanol intoxication. BMC Nephrol..

[79] Hantson P., Mahieu P. (2000). Pancreatic injury following acute methanol poisoning. J. Toxicol. Clin. Toxicol..

[80] Grufferman S., Morris D., Alvarez J. (1985). Methanol poisoning complicated by myoglobinuric renal failure. Am. J. Emerg. Med..

[81] Verhelst D., Moulin P., Haufroid V. (2004). Acute renal injury following methanol poisoning: analysis of a case series. Int. J. Toxicol..

[82] Cascallana J.L., Gordo V., Montes R. (2012). Severe necrosis of oesophageal and gastric mucosa in fatal methanol poisoning. Forensic Sci. Int..

[83] Akhgari M., Panahianpour M.H., Bazmi E. (2013 Mar). Fatal methanol poisoning: features of liver histopathology. Toxicol. Ind. Health.

[84] Kearney J., Rees S., Chiang W.K. (1997). Availability of serum methanol and ethylene glycol levels: a national survey. J. Toxicol. Clin. Toxicol..

[85] Blomme B., Lheureux P., Gerlo E., Maes V. (2001). Cobas Mira S endpoint enzymatic assay for plasma formate. J. Anal. Toxicol..

[86] Vinet B. (1987). An enzymic assay for the specific determination of methanol in serum. Clin. Chem..

[87] Laakso O., Haapala M., Jaakkola P. (2001). FT-IR breath test in the diagnosis and control of treatment of methanol intoxications. J. Anal. Toxicol..

[88] Hovda K.E., Urdal P., Jacobsen D. (2005). Case report: increased serum formate in the diagnosis of methanol poisoning. J. Anal. Toxicol..

[89] Osterloh J.D., Pond S.M., Grady S., Becker C.E. (1986). Serum formate concentrations in methanol intoxication as a criterion for hemodialysis. Ann. Intern. Med..

[90] Jones G.R., Singer P.P., Rittenbach K. (2007). The relationship of methanol and formate concentrations in fatalities where methanol is detected. J. Forensic Sci..

[91] Kostic M.A., Dart R.C. (2003). Rethinking the toxic methanol level. J. Toxicol. Clin. Toxicol..

[92] Shahangian S., Ash K.O. (1986). Formic and lactic acidosis in a fatal case of methanol intoxication. Clin. Chem..

[93] Hovda K.E., Julsrud J., Øvrebø S. (2011). Studies on ethylene glycol poisoning: one patient—154 admissions. Clin. Toxicol..

[94] Hoffman R.S., Smilkstein M.J., Howland M.A., Goldfrank L.R. (1993). Osmol gaps revisited: normal values and limitations. J. Toxicol. Clin. Toxicol..

[95] Haviv Y.S., Rubinger D., Zamir E., Safadi R. (1998). Pseudo-normal osmolal and anion gaps following simultaneous ethanol and methanol ingestion. Am. J. Nephrol..

[96] Schelling J.R., Howard R.L., Winter S.D., Linas S.L. (1990). Increased osmolal gap in alcoholic ketoacidosis and lactic acidosis. Ann. Intern. Med..

[97] Krahn J., Khajuria A. (2006). Osmolality gaps: diagnostic accuracy and long-term variability. Clin. Chem..

[98] Hovda K.E., Hunderi O.H., Rudberg N. (2004). Anion and osmolal gaps in the diagnosis of methanol poisoning: clinical study in 28 patients. Intensive Care Med..

[99] Jacobsen D., Bredesen J.E., Eide I., Ostborg J. (1982). Anion and osmolal gaps in the diagnosis of methanol and ethylene glycol poisoning. Acta Med. Scand..

[100] Lynd L.D., Richardson K.J., Purssell R.A. (2008). An evaluation of the osmole gap as a screening test for toxic alcohol poisoning. BMC Emerg. Med..

[101] Sanaei-Zadeh H. (2012). Response to “Methanol and ethylene glycol acute poisonings-predictors of mortality. Clin. Toxicol..

[102] Hassanian-Moghaddam H., Pajoumand A., Dadgar S.M., Shadnia S.H. (2007). Prognostic factors in methanol poisoning. Hum. Exp. Toxicol..

[103] Desai T., Sudhalkar A., Vyas U., Khamar B. (2013). Methanol poisoning—predictors of visual outcomes. JAMA Ophthalmol.

[104] Barceloux DG, Bond GR, Krenzelok EP, et al. American academy of clinical toxicology ad hoc committee on the treatment guidelines for methanol poisoning (2002) American academy of clinical toxicology practice guidelines on the treatment of methanol poisoning. J. Toxicol. Clin. Toxicol. 40 (4):415–446. 10.1081/clt-120006745 12216995

[105] Hovda K.E., Hunderi O.H., Tafjord A.B. (2005). Methanol outbreak in Norway 2002–2004: epidemiology, clinical features and prognostic signs. J. Intern. Med..

[106] Jacobsen D., Jansen H., Wiik-Larsen E. (1982). Studies on methanol poisoning. Acta Med. Scand..

[107] Liu J.J., Daya M.R., Carrasquillo O., Kales S.N. (1998). Prognostic factors in patients with methanol poisoning. J. Toxicol. Clin. Toxicol..

[108] Paasma R., Hovda K.E., Tikkerberi A., Jacobsen D. (2007). Methanol mass poisoning in Estonia: outbreak in 154 patients. Clin Toxicol Phila.

[109] Swartz R.D., Millman R.P., Billi J.E. (1981). Epidemic methanol poisoning: clinical and biochemical analysis of a recent episode. Med Baltim.

[110] Mathieu P., Hassoun A., Lauwerys R. (1989). Predictors of methanol intoxication with unfavourable outcome. Hum. Toxicol..

[111] Naraqi S., Dethlefs R.F., Slobodniuk R.A., Sairere J.S. (1979). An outbreak of acute methyl alcohol intoxication. Aust. N. Z. J. Med..

[112] Shadnia S., Rahimi M., Soltaninejad K., Nilli A. (2013 Oct). Role of clinical and paraclinical manifestations of methanol poisoning in outcome prediction. J. Res. Med. Sci.: the official journal of Isfahan University of Medical Sciences.

[113] Hantson P., Mahieu P. (2000). Pancreatic injury following acute methanol poisoning. J. Toxicol. Clin. Toxicol..

[114] Bennett I.L., Nation T.C., Olley J.F. (1952). Pancreatitis in methyl alcohol poisoning. J. Lab. Clin. Med..

[115] Eckfeldt J.H., Kershaw M.J. (1986). Hyperamylasemia following methyl alcohol intoxication. Source and significance. Arch. Intern. Med..

[116] Lazzeri C., Tarquini R., Giunta F., Gensini G.F. (2009). Glucose dysmetabolism and prognosis in critical illness. Intern Emerg Med.

[117] McCowen K.C., Malhotra A., Bistrian B.R. (2001). Stress-induced hyperglycemia. Crit. Care Clin..

[118] Sanaei-Zadeh H., Emamhadi M., Farajidana H. (2013 Jun 1). Electrocardiographic manifestations in acute methanol poisoning cannot predict mortality. Arh. Hig. Rada. Toksikol..

[119] Coulter C.V., Ibister G.K., Duffull S.B. (2011). The pharmacokinetics of methanol in the presence of ethanol. Clin. Pharmacokinet..

[120] Pietruszko R. (1975). Human liver alcohol dehydrogenase inhibition of methanol activity by pyrazole, 4-methylpyrazole,4-hydroxymethylpyrazole and4-carbopyrazole. Biochem. Pharmacol..

[121] Roy M., Bailey B., Chalut D. (2003). What are the adverse effects of ethanol used as an antidote in the treatment of suspected methanol poisoning in children?. J. Toxicol. Clin. Toxicol..

[122] Wedge M.K., Natarajan S., Johanson C. (2012). The safety of ethanol infusions for the treatment of methanol or ethylene glycol intoxication: an observational study. CJEM.

[123] Brent J., McMartin K., Phillips S. (2001). Fomepizole for the treatment of methanol poisoning. N. Engl. J. Med..

[124] Lepik K.J., Levy A.R., Sobolev B.G. (2009). Adverse drug events associated with the antidotes for methanol and ethylene glycol poisoning: a comparison of ethanol and fomepizole. Ann. Emerg. Med..

[125] Sivilotti M.L.A. (2008). Ethanol: tastes great! Fomepizole: less filling!. Ann. Emerg. Med..

[126] Boyer E.W., Mejia M., Woolf A., Shannon M. (2001). Severe ethylene glycol ingestion treated without hemodialysis. Pediatrics.

[127] Green R. (2007). The management of severe toxic alcohol ingestions at a tertiary care center after the introduction of fomepizole. Am. J. Emerg. Med..

[128] Anseeuw K., Sabbe M.B., Legrand A. (2007). Methanol poisoning: the duality between “fast and cheap” and “slow and expensive. Eur. J. Emerg. Med..

[129] Lepik K.J., Brubacher J.R., DeWitt C.R. (2008). Bradycardia and hypotension associated with fomepizole infusion during hemodialysis. Clin. Toxicol..

[130] Bestic M., Blackford M., Reed M. (2009). Fomepizole: a critical assessment of current dosing recommendations. J. Clin. Pharmacol..

[131] Brent J., Lucas M., Kulig K., Rumack B. (1991). Methanol poisoning in a 6-week-old infant. J. Pediatr..

[132] Marraffa J., Forrest A., Grant W. (2008). Oral administration of fomepizole produces similar blood levels as identical intravenous dose. Clin. Toxicol..

[133] Ghannoum M., Haddad H.K., Lavergne V. (2010). Lack of toxic effects of methanol in a patient with HIV. Am. J. Kidney Dis..

[134] Sanaei-Zadeh H., Zamani N., Shahmohammadi F. (2011). Can fomepizole be substituted by abacavir in the treatment of methanol poisoning?. J. Med. Toxicol..

[135] El-Bakary A.A., El-Dakrory S.A., Attalla S.M. (2010). Ranitidine as an alcohol dehydrogenase inhibitor in acute methanol toxicity in rats. Hum. Exp. Toxicol..

[136] Daugirdas J.T., Blake P.G., Ing T.S. (2015). Handbook of Dialysis.

[137] Rozenfield R.A., Leikin J.B. (2007). Severe methanol ingestion treated successfully without hemodialysis. Am. J. Therapeut..

[138] Hovda K.E., Jacobsen D. (2008). Expert opinion: fomepizole may ameliorate the need for hemodialysis in methanol poisoning. Hum. Exp. Toxicol..

[139] Hantson P., Haufroid V., Wallemacq P. (2005). Formate kinetics in methanol poisoning. Hum. Exp. Toxicol..

[140] Hovda K.E., Andersson K.S., Urdal P., Jacobsen D. (2005). Methanol and formate kinetics during treatment with fomepizole. Clin. Toxicol..

[141] Hovda K.E., Mundal H., Urdal P. (2007). Extremely slow formate elimination in severe methanol poisoning: a fatal case report. Clin. Toxicol..

[142] Pizon A.F., Brooks D.E. (2006). Hyperosmolality: another indication for hemodialysis following acute ethylene glycol poisoning. Clin. Toxicol..

[143] Jacobsen D., Ovrebo S., Sejersted O.M. (1983). Toxicokinetics of formate during hemodialysis. Acta Med. Scand..

[144] Jacobsen D., Webb R., Collins T.D., McMartin K.E. (1988). Methanol and formate kinetics in late diagnosed methanol intoxication. Med Toxicol Adverse Drug Exp.

[145] Kerns W., Tomaszewski C., McMartin K. (2002). Formate kinetics in methanol poisoning. J. Toxicol. Clin. Toxicol..

[146] Hirsch D.J., Jindal K.K., Wong P., Fraser A.D. (2001). A simple method to estimate the required dialysis time for cases of alcohol poisoning. Kidney Int..

[147] Zakariaei Z. (2020). Interleukin-10 may have diagnostic value in identifying mild traumatic brain injury. Brain Inj..

[148] Shadfar F., Zakariaei Z., Ghasempoori S.K. (2019). Effect of chelation therapy on lead-induced hepatotoxicity: a case series. Int. J. Med. Toxicol. Forensic Med..

[149] Zakariaei Z., Taslimi S., Tabatabaiefar M.A., Dargahi M.A. (2012). Bilateral dislocation of temporomandibular joint induced by haloperidol following suicide attempt: a case report. Acta Med. Iran..

[150] Youssef G.M., Hirsch D.J. (2005). Validation of a method to predict required dialysis time for cases of methanol and ethylene glycol poisoning. Am. J. Kidney Dis..

[151] Gilbert C., Baram M., Marik P.E. (2010). Continuous venovenous hemodiafiltration in severe metabolic acidosis secondary to ethylene glycol ingestion. South. Med. J..

[152] Roberts D.M., Yates C., Megarbane B. (2015 Feb 1). Recommendations for the role of extracorporeal treatments in the management of acute methanol poisoning: a systematic review and consensus statement. Crit. Care Med..

[153] Teo S.K., Lo K.L., Tey B.H. (1996). Mass methanol poisoning: a clinico-biochemical analysis of 10 cases. Singap. Med. J..

[154] Sharma R., Marasini S., Sharma A.K. (2012). Methanol poisoning: ocular and neurological manifestations. Optom. Vis. Sci..

[155] Marc-Aurele J., Schreiner G.E. (1960). The dialysance of ethanol and methanol: a proposed method for the treatment of massive intoxication by ethyl or methyl alcohol. J. Clin. Invest..

[156] Noker P.E., Eells J.T., Tephly T.R. (1980). Methanol toxicity: treatment with folic acid and 5-formyl tetrahydrofolic acid. Alcohol Clin. Exp. Res..

[157] Noker P.E., Tephly T.R. (1980). The role of folates in methanol toxicity. Adv. Exp. Med. Biol..

[158] Jacobsen D., McMartin K.E. (1986). Methanol and ethylene glycol poisonings: mechanism of toxicity, clinical course, diagnosis and treatment. Med. Toxicol..

[159] Liesivuori J., Savolainen H. (1991). Methanol and formic acid toxicity: biochemical mechanisms. Pharmacol. Toxicol..

[160] Pakravan M., Esfandiari H., Sanjari N. (2017 Apr 10). Additive effect of erythropoietin on conventional treatment of methanol induced toxic optic neuropathy. Bina Journal of Ophthalmology.

[161] Meyer R.J., Beard M.E.J., Ardagh M.W., Henderson S. (2000). Methanol poisoning. NZ Med J.

[162] Shukla M., Shikoh I., Saleem A. (2006). Intravenous methylprednisolone could salvage vision in methyl alcohol poisoning. Indian J. Ophthalmol..

[163] Abrishami M., Khalifeh M., Shoayb M., Abrishami M. (2011). Therapeutic effects of high-dose intravenous prednisolone in methanol-induced toxic optic neuropathy. J. Ocul. Pharmacol. Therapeut..

[164] Sanaei-Zadeh H. (2012). What are the therapeutic effects of high-dose intravenous prednisolone in methanol-induced toxic neuropathy?. J. Ocul. Pharmacol. Therapeut..

[165] Sodhi P.K., Goyal J.L., Mehta P.K. (2001). Methanol-induced optic neuropathy: treatment with intravenous high dose steroids. Int. J. Clin. Pract..

[166] Shah S., Pandey V., Thakore N., Mehta I. (2012). Study of 63 cases of methyl alcohol poisoning (hooch tragedy in Ahmedabad). J. Assoc. Phys. India.

[167] Skolnik A.B., O'Connor A., Ruha A.M., Curry S. (2012). Recommendations regarding management of methanol toxicity. Ann. Emerg. Med..

[168] Konishi Y., Chui D.H., Hirose H. (1993). Trophic effect of erythropoietin and other hematopoietic factors on central cholinergic neurons in vitro and in vivo. Brain Res..

[169] Ashwal S., Cole D.J., Osborne S. (1995). A new model of neonatal stroke: reversible middle cerebral artery occlusion in the rat pup. Pediatr. Neurol..

[170] Feng Q. (2006). Beyond Erythropoiesis: the Anti-inflammatory Effects of Erythropoietin.

[171] Gorio A., Gokmen N., Erbayraktar S. (2002). Recombinant human erythropoietin counteracts secondary injury and markedly enhances neurological recovery from experimental spinal cord trauma. Proc. Natl. Acad. Sci. Unit. States Am..

[172] Katavetin P., Tungsanga K., Eiam-Ong S. (2007). Antioxidative effects of erythropoietin. Kidney Int..

[173] Grasso G. (2001). Neuroprotective effect of recombinant human erythropoietin in experimental subarachnoid hemorrhage. J. Neurosurg. Sci..

[174] Pakdel F., Sanjari M.S., Naderi A. (2018). Erythropoietin in treatment of methanol optic neuropathy. J. Neuro Ophthalmol..

[175] Belson M., Morgan B.W. (2004). Methanol toxicity in a newborn. Clin. Toxicol..

[176] Caravati E.M., Erdman A.R., Christianson G. (2005). Ethylene glycol exposure: an evidence-based consensus guideline for out-of-hospital management. Clin. Toxicol..

[177] Brent J. (2010). Fomepizole for the treatment of pediatric ethylene and diethylene glycol, butoxyethanol, and methanol poisonings. Clin. Toxicol..

[178] Loza R., Rodriguez D. (2014 Jan 6). A case of methanol poisoning in a child. Case reports in nephrology.

